# Investigation of the Effects of Phosphate-Containing Flame-Retardant Chemicals on Particleboards Produced Using Urea–Formaldehyde Resin

**DOI:** 10.1021/acsomega.4c03419

**Published:** 2024-09-14

**Authors:** Nazan Uygur, Erdinc Doganci, Ayse Aytac

**Affiliations:** † Department of Polymer Science and Technology, Kocaeli University, 41001 Kocaeli, Turkey; ‡ Department of Chemistry and Chemical Processing Technology, Kocaeli University, 41140 Kocaeli, Turkey; § Department of Chemical Engineering, Kocaeli University, 41380 Kocaeli, Turkey

## Abstract

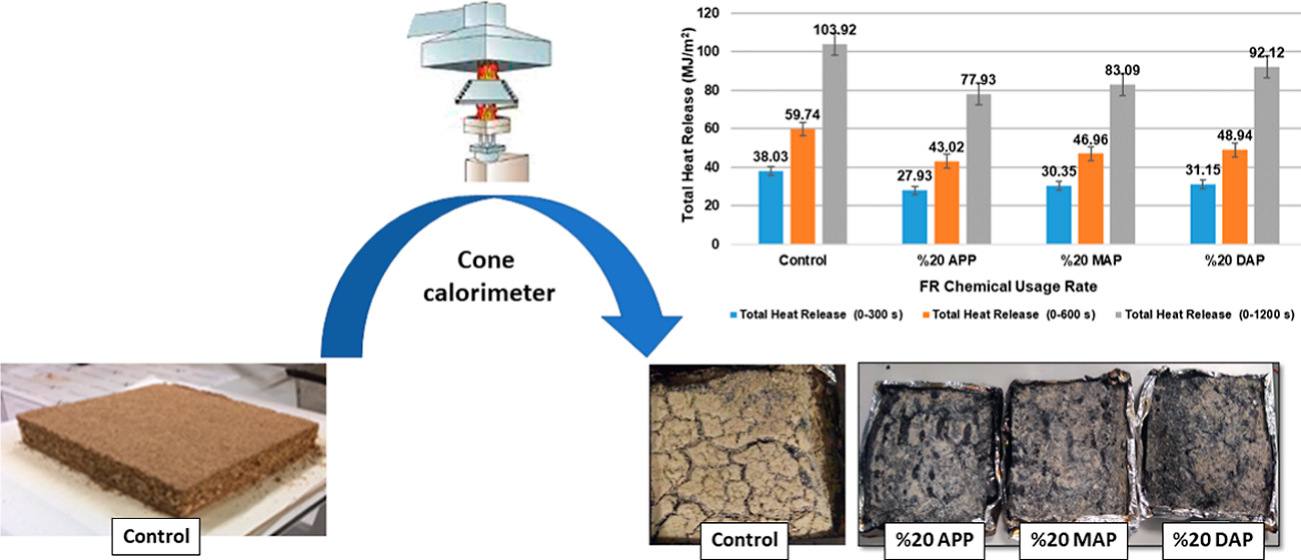

In this study, urea–formaldehyde resins containing different phosphate-based flame retardants (FRs) were prepared to produce wood-based panels (medium-density fiberboard, particleboard, and veneer board), and their flammability properties were investigated. The changes in the gelation times were investigated when the phosphate-containing FRs used in different amounts were mixed with the resin. Chemicals that have both hardener and flame-retardant properties at the same time have been studied. In addition, the pH and thermal analyses of the resin and flame-retardant mixtures prepared in this study were performed by a pH meter and thermogravimetric analysis, respectively. The limiting oxygen index, UL-94 vertical burning, and cone calorimetry tests on the samples with the best combustion characteristics were carried out by sizing the particleboards in accordance with the standards and applying the prepared flame-retardant resins on them. The distribution of flame-retardant resin on the particleboard surface was investigated for the samples prepared by scanning electron microscopy. As a result of the analyses, it was determined that the samples containing 20% ammonium polyphosphate had good nonflammability properties.

## 1 Introduction

Wood and its derivatives constitute a significant natural resource that plays a pivotal role in human existence. These materials, with their myriad applications, are integral to the production of items such as particleboard, furniture, paper, parquet flooring, musical instruments, wire posts, coated boards, plywood, and medium-density fiberboard. The appeal of wood lies in its hardness, durability, flexibility, lightness relative to its strength, malleability, screw retention, and potential for property enhancement. The extensive utilization of wood materials can be attributed to their physical and mechanical properties, structural characteristics, and chemical components.^1−3^ One of the primary drawbacks of wood materials is likely their easy combustibility, which restricts their widespread application in both residential and nonresidential construction.^4−6^ To date, the combustion behavior of wood has been complex. However, the mechanisms behind ignition, pyrolysis, extinction, and combustion are well-understood in the fire science literature over a wide range of experiment conditions for key parameters such as critical heat of combustion and flux for ignition. When exposed to heat, the natural polymers in wood undergo decomposition, resulting in the production of inert and combustible gases, inorganic ash, solid carbonaceous char, and liquid tars. Upon heating, and before the initiation of pyrolysis, free water also begins to evaporate as temperatures within the wood material approach 100 °C. In some cases, water vapor may penetrate further into the sample and move away from the heat source.^7−9^

One of the methods used to impart nonflammability to particleboard, whose raw material is wood, is to use various flame retardant (FR) chemicals in the resin used in particleboard making. However, due to increasing environmental concerns, the utilization of effective halogen-based FRs has been restricted in both the wood and plastics industries. Halogenated (chlorinated and brominated) FRs have some drawbacks including the potential to erode metal components, a tendency to bioaccumulate, and the ability to produce corrosive hydrogen halides and toxic gas during combustion.^10−14^ Furthermore, halogenated FRs often need toxicologically critical antimony oxides as synergists. Halogen-free FRs have been demonstrated to be safer for both humans and the environment, even though halogenated FRs are very good at suppressing flammability. Phosphorus, inorganic, and nitrogen (PIN)-containing compounds are halogen-free alternative FRs, the so-called PIN FRs. The use of FR chemicals results in a reduction in the intensity of combustion during the initial stages of a fire, thereby reducing the emission of gases. They do not contribute to the toxicity of smoke or the corrosiveness of gases, and they are also known to result in a lower smoke density. Consequently, several international regulations have been enacted that require the utilization of PIN FRs in items crafted from wood in a multitude of sectors. The role of phosphorus and nitrogen compounds in carbon degradation has received increased attention in both industrial and academic contexts. These compounds can result in carbon-yielding reactions in place of the typical outcome of carbon degradation, namely, the formation of carbon monoxide.^15−20^ Phosphorus-containing compounds are the most widely utilized FRs for wood and wood-based products. Monoammonium phosphate (MAP) and diammonium phosphate (DAP), ammonium salts with phosphorus in their structures, are the most preferred flame-retardant compounds due to their easy application properties to wood and their high effectiveness. The FR processes applied to wood products have been devised to decrease their flammability. Pressure or soaking impregnation are the most popular techniques for mixing FRs in plywood or wood products.^21,22^

Glues used in the production of particleboards are used under a hot press and at a certain pressure and time to stabilize chips and flakes to the desired thickness. In the particleboard industry, melamine–urea–formaldehyde, urea–formaldehyde (UF), resorcinol–formaldehyde, and phenol–formaldehyde resins, often called thermoset resins, are used. UF resin was developed in the 1930s and was developed as a binder for particleboard, and today, approximately 90% of the particleboards produced globally are manufactured using UF resin.^23−26^ In addition, since MF resins are more water-resistant than UF resins, they have been extensively employed in areas where the product may potentially come into contact with water, such as kitchen furnishings and exterior-grade panel items. The only drawback is the higher cost of MF resin than that of UF resin. Thermoset resins first soften when heated but harden when heated further, so as not to soften again. UF resins are the most preferred adhesive resins in particleboard production today due to the short hardening time in hot pressing processes, ease of use, and low cost. Due to their white color and transparent structure, they are used in particleboards produced for general purposes.^27−33^

The objective of this study is to enhance the flame resistance of particleboards that exhibit a high tendency to combustion. For this purpose, the mixtures prepared by adding solid flame-retardant chemicals to the UF resin used in the production of particleboards were applied to the particleboard samples. As far as we know, there is no study in the literature that examines and compares the effect of selected FRs in the resin used in particleboard production. The pH and curing (gel) times of the prepared mixtures were checked. Concurrently, the impact of phosphate-containing FRs incorporated during the curing process on the thermal properties of the UF resin was evaluated through thermogravimetric analysis (TGA). Limiting oxygen index (LOI), UL-94, and cone calorimetry tests were used to determine the flame resistance of the samples prepared during the study. The surface morphologies of the samples prepared by scanning electron microscopy (SEM)–energy dispersion spectrometry (EDX) were also examined. In this study, solid FR chemicals with both curing and flame-retardant properties were used. It is aimed to produce the best flame-retardant particleboard with less cost.

## 2 Materials and Experiments

### 2.1 Materials

Ammonium polyphosphate (APP) (Uniform, decomposition temperature >285 °C, Bulk density: 700 kg/m^3^), DAP (Merck, decomposition temperature: 155 °C, 50 g/L (at 20 °C) pH: 7.8–8.5), MAP [Merck, melting point: 190 °C, density (at 20 °C): 1.8 g/cm^3^] were used as the FR materials in this study. The UF resin used is 65% UF resin obtained from Yıldız Entegre’s Kocaeli/Turkey.

### 2.2 Methods

In the study, UF resins with and without FRs have been prepared. The pH and gel times of these mixtures were examined and cured in the oven. For LOI, UL-94, cone calorimetry, and SEM analyses, 8 and 18 mm particle boards produced in Yıldız Entegre’s Akhisar plant were used. UF resin and UF resin with FR compounds were applied to both the bottom and top surfaces of the particleboard, which was sized to the desired dimensions, and analyzed after drying in the oven. No chemicals were added to the 65% UF resin for the control sample. APP, MAP, and DAP FR chemicals were mixed into 50 g of resin weighing 5%, 10%, 15%, and 20% solids by calculating 65% solids. The pH and gel times of the prepared mixtures were primarily measured. Then, after applying a brush to the upper and lower surfaces of the particleboards cut to the size and thickness specified in the standards at a rate of 250 g/m^2^, they were dried slowly in the oven at 23 ± 2 °C and 50% ± 5 relative humidity for 24 h. The prepared particleboard samples were then subjected to LOI, UL-94, and cone calorimetry tests. At the same time, the prepared resin mixtures are cured in the oven for thermal analysis via TGA. Also, SEM images were taken to examine surface morphologies.

### 2.3 Physical Properties

pH and curing (gel) time analyses of the plates were carried out in the Başiskele Yıldız Integrated Glue Plant quality control laboratory. 50 g of 65% UF resin was weighed. For the control sample, the pH of the resin was measured at 25 °C in the Mettler Toledo brand pH meter, which is a tabletop type, without adding any flame-retardant chemicals. Then, 50 g of UF resin was weighed in four different beakers, and APP, MAP, and DAP chemicals were added with 5%, 10%, 15%, and 20% of the solid amount based on the 65% solids basis of the resin. The pH of the mixtures was measured at 25 °C.

Mixtures prepared for pH analyses were used for the gel time analysis. A 3 g sample of the mixtures was added to the test tube in accordance with the TS EN 12009 standard, and the stopwatch was operated as soon as the test tube was immersed in boiling water at 100 °C. The resin sample inside the test tube was continuously stirred until gelling was observed in the glue, and the stopwatch was stopped at the moment when the mixing became difficult, i.e., when the resin hardened. Gel times for the UF resins have been identified.

### 2.4 Thermal and Morphological Properties

Thermal (TGA) and surface morphology (SEM) analyses were performed to observe the effect of FR-added resins on flame retardancy. 65% UF resin with no FR chemicals added and UF resin mixed with FR chemicals were cured in a 120 °C oven, and thermal analysis was performed using the Mettler Toledo device. The samples, which weighed approximately 2.0–2.5 mg, were heated in ceramic containers in a nitrogen atmosphere at a rate of 10 °C/min from 25 to 600 °C and cooled rapidly. Thus, it was observed how much % mass was lost with the temperature increase from the thermal degradation curves of resins with FR added. Samples were prepared for the LOI test, and UL-94 was overprepared and used to study surface morphologies. For the surface morphology of the prepared samples, a scanning electron microscope was made with 500×1000× and 2000× magnification in the NABİLTEM Laboratory at Tekirdağ Namık Kemal University, and the obtained data were recorded.

### 2.5 Combustion Analyzes

UL-94 vertical burning, LOI, and cone calorimetry tests were applied to the clipboard samples. The prepared resins with FR chemicals were applied with a brush of 250 g/m^2^ on the lower and upper surfaces of particleboards sized to 80 × 1 × 8 mm and dried in the oven set to 25 °C for 24 h. To measure the flame resistance of particle boards, analyses were performed in accordance with the ISO 4589-2 standard in the LOI test device. The combination of oxygen and nitrogen upward through the wooden samples is placed vertically with the mold to hold it in a glass chimney. The upper end of wood particleboard samples is exposed to flame. After the boards were ignited, the combustion behavior of the samples was examined, and the number of seconds the burning lasted and the length of the burning wood particleboard sample were compared with the previously determined values for combustion. The results were also recorded by finding the volume in % oxygen.

The UL-94 vertical combustion test was carried out on the prepared samples in line with the standard set forth by ASTM D 3801-0. The samples are fixed perpendicular to the holder, and the blue flame is set to be 20 ± 2 mm high. The samples were perpendicular to the blue flame for 10 s. The flame extinction time was recorded as *t*_1_, and the same sample was again caught in flame for 10 s after the extinction event was over. This postflame extinction time was also recorded as *t*_2_. At the same time, it was also observed that the cotton in the lower part of the system fell on the flame after exposure to the flame during the test and whether the dropped droplet ignited the cotton. Then, one by one, all samples were tested, and their effects against flame were recorded.

The control sample and the prepared UF resins with FR chemicals were applied to the upper and lower surfaces of the particle boards sized in dimensions of 100 × 100 × 18 mm with a brush of 250 g/m^2^. Then, the particle boards were dried slowly in the oven at 23 ± 2 °C and at %50 ± 5 relative humidity for 24 h. Four of the prepared particleboards were produced for cone calorimeter testing according to the TS ISO 5660-1. The cone calorimetry analysis allowed the observation of the heat release, oxygen consumption, and smoke formation of the samples.

## 3 Results and Discussion

### 3.1 Physical Features

The pH values of the mixtures prepared with different FRs and ratios after mixing with UF resin with an average pH value of 8.4 are given in Table 1. Besides, the gel times determined in accordance with the TS EN 12009 standard from the prepared mixtures are given in Table 1. The lower the pH value is of the mixtures, the faster the hardening that occurs with the temperature. The highest pH and gel time changes were observed in the mixtures in which MAP was used as the FR chemical. The pH values of the mixtures with DAP were not altered significantly. But the gel time values were very high, and slower hardening occurred. The closest values to the gel time of the UF control sample were obtained when APP was used in UF. pH control is very important in the sheet manufacturing process as it has an impact on the hardening time of the glue. In plate production, a sufficient pressing time is calculated according to the gel times of the resin. The hardening of the UF resin is further accelerated by increasing the temperature. Changes in the pH value affect the gelling time.

**Table 1:** pH Measurements and Gel (Curing) Times of Samples

FR	sample	amount of FR (%)	amount of FR (g)	pH	gel time (s)
neat UF	control	no	no	8.50	43
UF/APP	1	5	1.62	5.71	57
	2	10	3.25	5.73	53
	3	15	4.87	5.85	51
	4	20	6.50	5.85	51
UF/MAP	5	5	1.62	5.15	31
	6	10	3.25	5.06	29
	7	15	4.87	5.05	28
	8	20	6.50	5.05	28
UF/DAP	9	5	1.62	7.60	90
	10	10	3.25	7.85	125
	11	15	4.87	7.90	not jelled
	12	20	6.50	8.02	not jelled

### 3.2 Thermogravimetric Analysis

**Figure 1:**
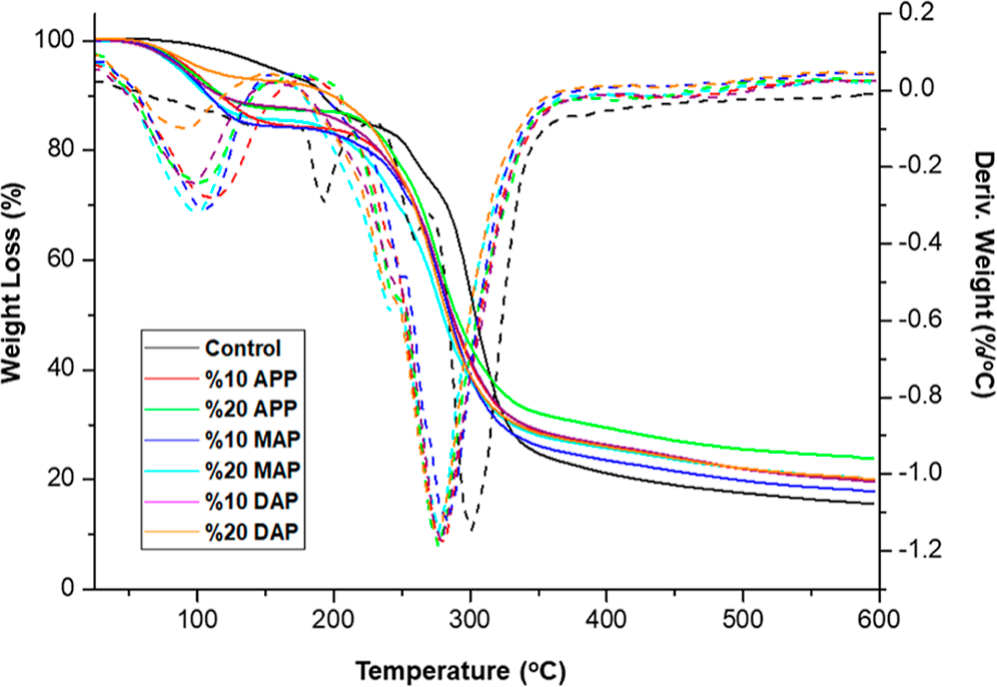
TGA thermograms of samples.

Thermal stability of the prepared UF resins with and without FR was examined by using TGA. Table 2 summarizes the results on their onset (*T*_d,onset_), *T*_10_, *T*_50_, and maximum (*T*_d,max_) thermal breakdown temperatures, as well as their char yields, whereas Figure 1 depicts their thermograms.

**Table 2:** Thermal Properties of UF Resins

material	*T*_d_,_onset_ (°C)^a^	*T*_10_ (°C)^a^	*T*_50_ (°C)^a^	*T*_d_,_max_ (°C)^b^	char yield (%)^c^
control (neat UF)	151	190	304	301	15.6
%10 APP	94	118	286	280	19.7
%20 APP	91	118	287	277	23.7
%10 MAP	87	108	284	283	17.8
%20 MAP	87	106	280	277	20.0
%10 DAP	91	117	287	282	19.4
%20 DAP	104	200	286	279	19.9

a *T*_d_,_onset_, *T*_10_, and *T*_50_ are weight loss temperatures at the onset, 10%, and 50%, respectively.

b *T*_d_,_max_ is the temperature that corresponds to the highest rate of weight loss.

c The amount of mass % remaining at 600 °C.

According to TGA, the *T*_onset_, *T*_10_, *T*_50_, and *T*_max_ of the UF resins with fire retardants were lower than those of the control sample. On the contrary, it was detected that the percentage char yields of the FR-added resins were higher than that of the control sample. When the *T*_onset_ and *T*_10_ temperatures were investigated, it was observed that the lowest degradation temperatures belonged to the resins with MAP added. When *T*_50_ and *T*_max_ temperatures were examined, it was determined that the temperature values of all resins with FR added were almost close to each other.

### 3.3 Limit Oxygen Index

The polymers’ chemical structures have a major impact on the LOI values. The number of oxidizable atomic or molecular groups in polymers can be used to calculate LOI values. The materials’ flammability increases with an increase in the ratio of carbon and hydrogen, which determines flammability. A material with a high level of LOI is less prone to combustion when it is exposed to atmospheric conditions. As a result, materials having a lower LOI value than 25% are more likely to burn when exposed to air, whereas materials having a higher LOI value than 25% can self-extinguish in the same conditions.^34,35^

LOI measurements are a common method of evaluating the flame-resistant properties of chemically added wood materials that have been treated with FRs. LOI tests were carried out on all of the particleboard samples following the TS EN ISO 4589-2 standard. The LOI values of particle board samples are given in Table 3 in O_2_ %. According to the LOI results, as stated in Table 3, it was found that particleboard samples coated with 20% FR chemical-added resin had the highest flame resistance with 29.1% and 29.2% LOI values. It was found that there was a 16% enhancement in flame resistance in comparison to the control sample. The results demonstrate that the incorporation of FR chemicals enhances flame resistance in comparison with that of the control sample. It can be concluded that the study achieved its intended objective. In addition, each of the particle board samples did not exceed the 180 s combustion time limit specified in the standard and showed an “X response” by not exceeding the 50 mm combustion limit in the boards.

**Table 3:** LOI and the Amount of Combustion Values

FR	sample	amount of FR (%)	LOI (%)	burning time (s)	combustion condition
control	control	no	25.1	23	not passed
APP	1	5	27.4	14	not passed
	2	10	27.9	12	not passed
	3	15	28.6	10	not passed
	4	20	29.1	11	not passed
MAP	5	5	27.7	12	not passed
	6	10	27.9	14	not passed
	7	15	28.8	15	not passed
	8	20	29.2	16	not passed
DAP	9	5	27.7	10	not passed
	10	10	28.1	11	not passed
	11	15	28.5	16	not passed
	12	20	29.1	15	not passed

### 3.4 UL-94 Vertical Combustion Test

Samples that are positioned vertically and come into contact with a burner flame as an ignition source have their combustion behavior examined by using the UL-94 vertical combustion test. In this test, the samples are classified in three ways (V, V1, and V2) regarding the individual burning time of each sample, the material’s flammability, each sample’s total burning time, and the number of droplets that fall.^36^ This test was performed to determine the flammability of particleboard samples prepared by applying resins and the dispersibility of the flame when exposed to flame at low rates. The obtained UL-94 results are listed in Table 4. The control plate sample falls into the flammable material category with the combustion of more than 4 min, while particleboard samples with resin with FR chemicals are V-0 with their fast extinguishing time. APP, MAP, and DAP FR chemicals are thought to slow down the flame and keep the samples’ surfaces from coming into touch with oxygen, forming a charred layer in a way that shows preservative.^37^ To be evaluated as V-0, the particle boards containing APP, DAP, and MAP as FRs should not be seen to drip by sparking, and the cotton underneath should not be ignited by the spark. Particleboard samples prepared by adding different FR chemicals, including the control sample, did not spark and drip, and the cotton placed under the assembly from the beginning of the experiment was not ignited by the dripping of sparks. All the samples prepared showed similar properties, and the act of ignition of the cotton by any dripping did not occur.

**Table 4:** UL-94 Vertical Combustion Test of Chemicals Added in Different Ratios

FR	sample	amount of FR (%)	*t*_1_ (s)	*t*_2_ (s)	UL-94	dripping	flammability^a^
control	control	no	4 min	TBC^b^	FM^c^	no	no
APP	1	5	4	7	V_o_	no	no
	2	10	3	5	V_o_	no	no
	3	15	3	4	V_o_	no	no
	4	20	2	3	V_o_	no	no
MAP	5	5	5	8	V_o_	no	no
	6	10	3	6	V_o_	no	no
	7	15	2	5	V_o_	no	no
	8	20	2	3	V_o_	no	no
DAP	9	5	3	8	V_o_	no	no
	10	10	2	5	V_o_	no	no
	11	15	2	4	V_o_	no	no
	12	20	0	4	V_o_	no	no

a Ignition of cotton with drops.

b The burning continued.

c Flammable material.

### 3.5 Cone Calorimeter Test

One of the most significant bench-scale instruments for assessing the impact of adding FRs is the cone calorimeter.^38^ Four of the particleboards were analyzed by ISO 5660-1^39^ with a cone calorimetry device to determine the fire performance of the samples with FRs added with 20% of the solids ratio that gave the best results from flame resistance analyses of particleboard samples with FR-added resin mixture. The basic technique used to calculate the rate of heat release is based on observing the decreasing oxygen concentration in the combustion products of a known standard sample under a certain heat flux. Cone calorimetry provides a comprehensive understanding of the fire risk and hazard associated with a given sample. This includes an assessment of the ignition time, heat release rate (HRR), and total heat release (THR), as well as an evaluation of the oxygen consumption, CO production, and smoke production. The scientific understanding of the fire reaction and combustion behaviors of the samples is easily achieved with these test data.

The results obtained in the cone calorimetry test reveal very important information about the tendency and behavior of different materials against combustion. The average results of THR, HRR, effective heat of combustion (EHC), ignition time, mass loss rate, and specific extinction area values defined via the cone calorimetry studies are indicated in Table 5. The “specific extinction area,” a measurement of a material’s smoke generation, is typically used to describe smoke data from cone calorimeters.^40^ The ratio of the mass loss rate to the HRR in a small-scale calorimeter is known as the EHC.^41^ Consequently, the energy released per unit mass loss in the cone calorimetry test may be defined as the EHC. The mass loss is determined by measuring the initial mass of the specimen and then calculating the result using numerical means.^42^ The term “ignition time” is described as the time at which ignition occurs for the first time on the sample surfaces. This parameter is crucial for assessing the plate’s flame performance. The higher the ignition difficulty, the longer the ignition period, and the substance becomes more difficult to ignite. Therefore, the advantages of reducing flammability and fire hazard are provided by extending the ignition time of the processed samples.^43,44^ In comparison to the control sample, it was found that the time required for the particleboard sample containing 20% APP to ignite increased by 50%, 30% for the board containing 20% MAP, and 15% for the particleboard containing 20% DAP. This means that the sample containing 20% APP has the highest ignition time and thus has a higher combustion resistance. Table 5 shows the average results of the cone calorimetry.

**Table 5:** Cone Calorimetry Test Average Results of the Prepared Samples

parameter	control	%20 APP	%20 MAP	%20 DAP
ignition time (s)	20	30	26	23
heat release rate (1st peak HRR, kW/m^2^)	265.6	169.3	180.5	192.9
heat release rate (2nd peak HRR, kW/m^2^)	83.4	85.5	81.3	96.1
total heat release (THR, Mj/m^2^)	128.9	100	107.2	106.7
specific extinction area (m^2^/kg)	15.2	33.4	37.6	41.4
effective heat of combustion (EHC, MJ/kg)	12.6	11.3	12.1	11.9
mass loss rate (g/s m^2^)	6.94	5.91	5.92	6.74
average CO (kg/kg)	0.0197	0.0137	0.0113	0.139
average CO_2_ (kg/kg)	1.20	1.11	1.13	1.18
CO/CO_2_ ratio	0.016	0.012	0.010	0.117
smoke production rate (m^2^/s)	0.022	0.024	0.030	0.026
total smoke production (m^2^)	1.95	3.13	3.41	3.78
total smoke release (TSR, m^2^/m^2^)	220.7	312.7	341.5	378.1
maximum average heat release rate (MARHE, kW/m^2^)	176.5	120.0	128.4	135.7

**Figure 2:**
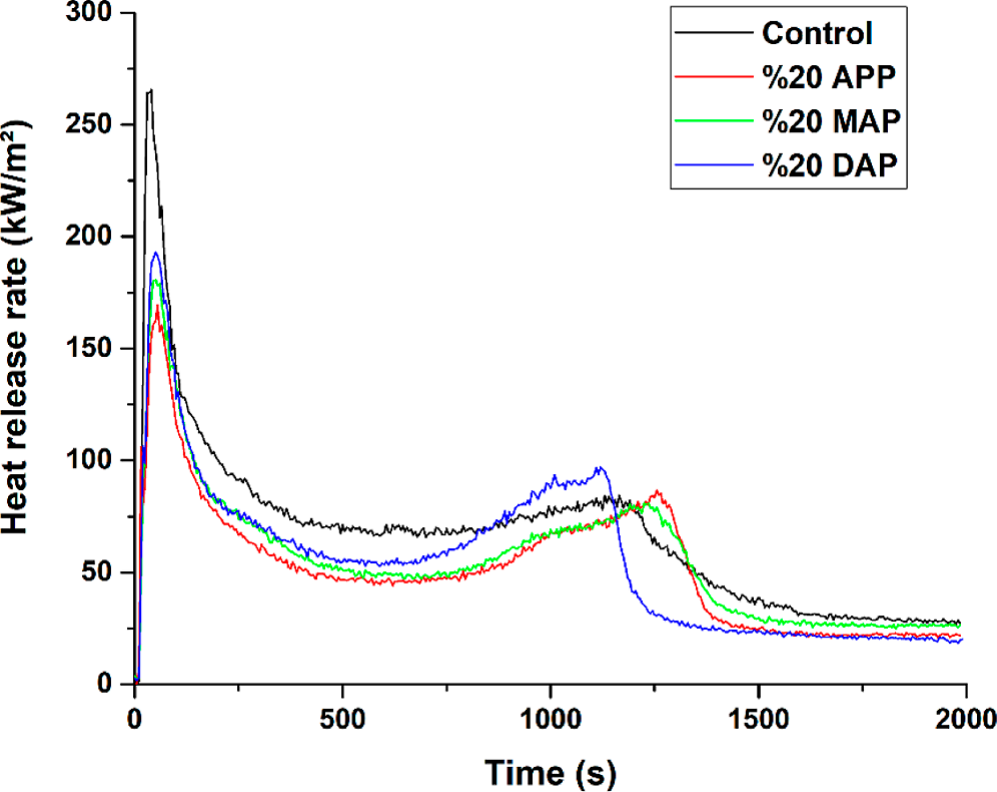
Comparative heat release graph of particleboard samples.

The parameters of smoke production rate, THR, and HRR are of significant importance in the assessment of the flame retardation and combustibility properties of interior materials.^45^ In the cone calorimetry test, the HRR is a significant parameter employed in the investigation of the combustion behavior of samples. The term “heat release”, which is determined by the amount of oxygen used during combustion, is the amount of heat released from the sample per unit of time.^46^ As can be seen in Figure 2, the added FR chemicals caused a decrease in the HRR values of the particleboards. Upon igniting samples, the HRR exhibited an initial increase, subsequently decreasing and then increasing again. The HRRs exhibited two exothermic peaks over time in all samples.^47−49^

It is reported that the combustion process exhibits two distinct peaks, the first occurring during the initial combustion phase and the second preceding the extinguishing of the flame.^47,48^ To decompose the flammable material, the wooden samples’ surface was first exposed to heat radiation. The first exothermic peak, mainly due to the formation of a charred layer on the surface, occurred as the sample continued to burn under high temperature conditions. The formation of this layer subsequently results in a reduction in the rate of heat release, which can be attributed to the outer charred layer. The outer charred layer serves to attenuate the rate and magnitude of gas and heat emissions from the inner combustion area. The particleboard samples were found to have contracted and bent following the initial peak in the heat release, while the coal layer that had developed on the particleboard surface had greatly increased in volume. The shrinking and bending particleboard has been reported to burn from the edges and bottom. The carbonized layer reduced the temperature and intensity of the sample combustion by enhancing the distance between the underlying materials and flame. At high temperatures, the heat accumulated behind the sample initiates the second exothermic peak.^50^ The heat is progressively transported to the inside of the space. The inside of the samples is then heated, decomposed, and burned at high temperatures. As the amount of combustible material increased and the rate of heat release increased, the second exothermic peak occurred.^51^ During the evaluation, the highest rates of heat emission were used. Examining the values of the first exothermic peak, the sample with the highest diminish is the particleboard sample with 20% APP FR. In comparison to the control sample, the particleboard sample containing 20% APP FR showed a 36% reduction in HRR. In particleboard samples with 20% MAP and 20% DAP FR chemical, the rate of heat release dropped by 32% and 27%, respectively.

**Figure 3:**
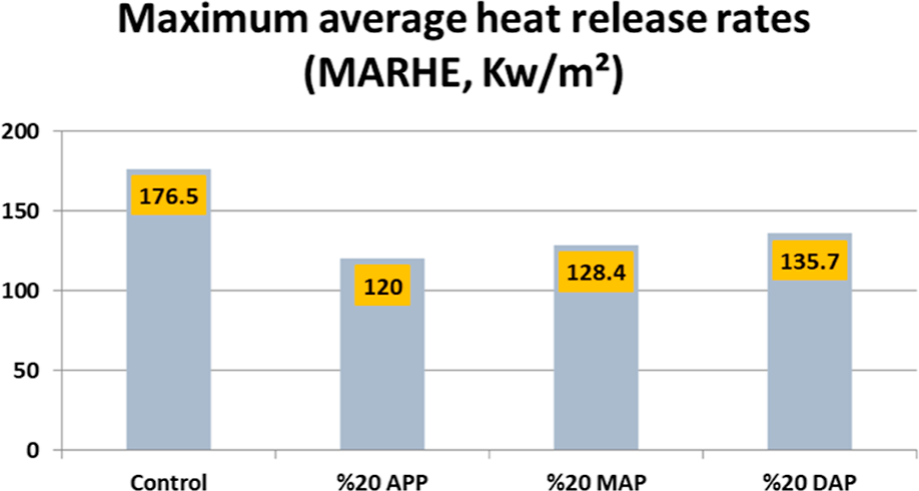
Comparison of maximum average HRR values of particleboard specimens.

Maximum average rate of heat emission (MARHE) values, which are a very useful parameter for predicting how large-scale combustion may develop, also show that combustion slows when the FR is added. As can be seen in Figure 3, the MARHE value for the control sample decreased from 176.5 to 120 kW/m^2^ when 20% APP was added, to 128.4 kW/m^2^ when 20% MAP was added, and to 135.7 kW/m^2^ when 20% DAP was added. APP, DAP, and MAP are three typical inorganic phosphorus FRs. Unlike others, APP is a linear or branched polymeric compound with a variable degree of polymerization (n). Generally, long-chain APP decomposes at temperatures above 300 °C, producing poly(phosphoric acid) and ammonia, while short-chain APP degrades at 150 °C. The choice of APP as a FR, therefore, depends largely on the processing temperature of the materials. When APP is added to polymeric substances, that material contains elements of oxygen and/or nitrogen, and the result can be the formation of charcoal. At elevated temperatures, APP undergoes decomposition, forming free acidic hydroxyl groups, ultraphosphate, and polyphosphoric acid. These compounds act as catalysts in the dehydration reaction of polymers, yielding char residues. Besides, APP only changes the polymer’s degradation mechanism in nonself-charring polymeric substances.^52−55^

**Figure 4:**
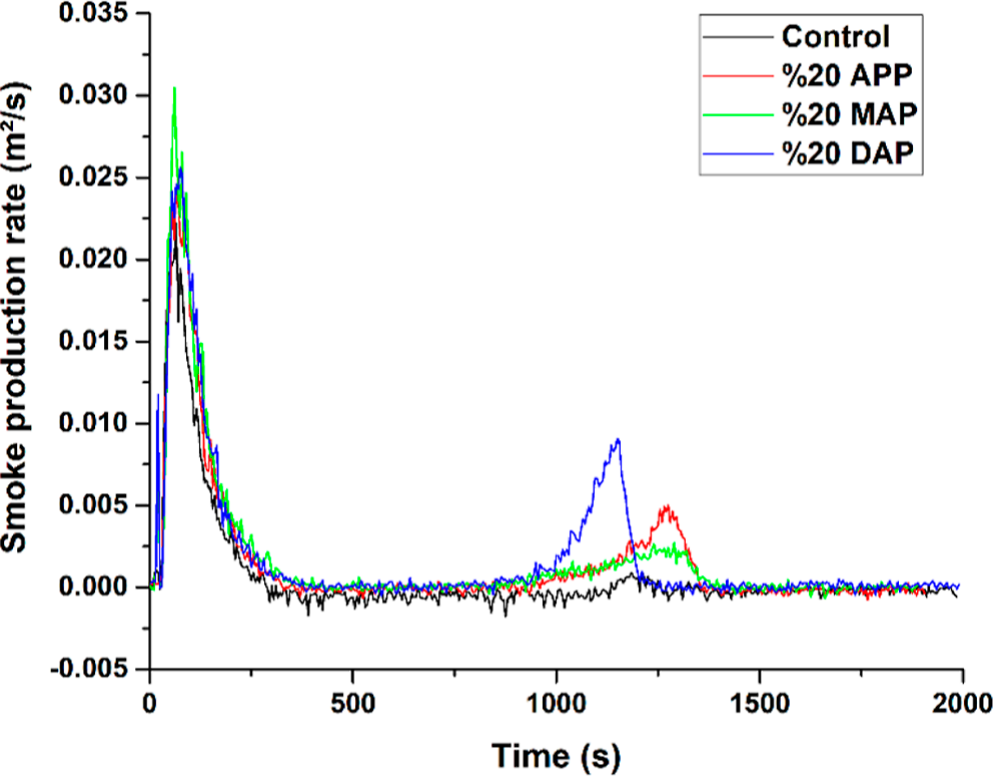
Comparison graph of particleboard sample smoke generation rates.

By dividing a given extinguishing area by the mass loss ratio, one may compute the smoke production rate, which is the amount of concentrated smoke emitted per unit of time. The overall amount of smoke that the material releases during the pyrolysis and combustion processes is known as total smoke production. The comparison of the particleboard samples’ smoke generation rates is displayed in Table 5 and Figure 4. In comparison to the control sample, the particleboard samples containing 20% APP, 20% MAP, and 20% DAP demonstrated an increase in smoke production rates of 9.1%, 36.4%, and 18.2%, respectively. In comparison with the control samples, 60.5%, 74.9%, and 93.8% enhancement were detected for the particleboard samples containing 20% APP, 20% MAP, and 20% DAP, respectively. Average CO and CO_2_ values also vary. The CO/CO_2_ ratio is used to evaluate the toxicity of the smoke generated during combustion. As this value increases, the toxicity of smoke will also increase. When the results obtained are evaluated, this value decreases with the addition of the FR, except for the particleboard sample containing 20% DAP.

**Figure 5:**
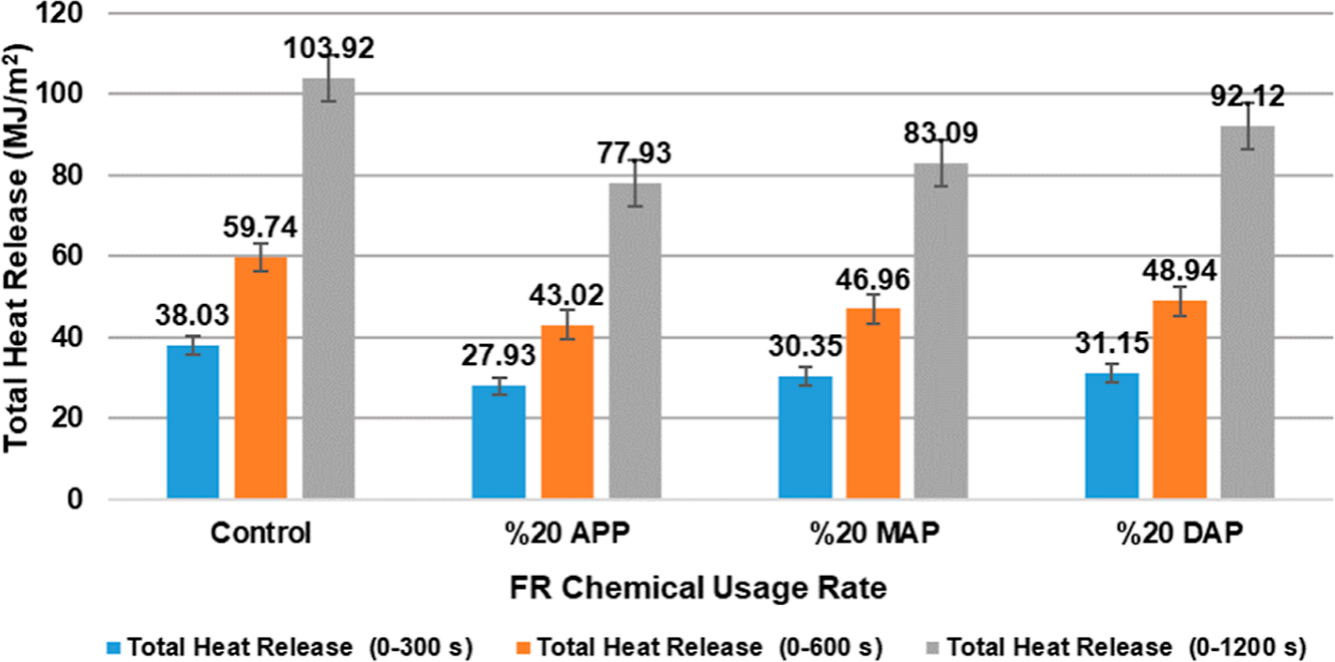
Total heat dissipation values of particleboard samples over a specified period.

Figure 5 and Table 5 display the particleboard samples’ THR values. Figure 5 shows the total heat dissipation (0–300) of the particleboard samples. In comparison to the control sample, it was found that samples comprising 20% APP, 20% MAP, and 20% DAP exhibited reductions of 26.55%, 20.19%, and 18.09%, respectively. The particleboard sample containing 20% APP shows the highest reduction.

The specific extinction area is another index of smoke emission. It represents a measure of smoke efficiency and indicates that, in general, the higher this value is, which represents smoke efficiency, the more smoke is released per kilogram of the sample.^56^ A significant increase was observed in the specific extinction values for samples containing 20% APP, 20% MAP, and 20% DAP FR, found to be more than double those of the control sample.

The energy released per unit mass lost by combustion is known as the EHC. When compared with the control sample in Table 5, all samples showed a decline in the EHC values. Particleboard samples containing 20% APP, 20% MAP, and 20% DAP yielded EHC values of 11.3, 12.1, and 11.9 MJ/kg, respectively. The results indicate that the volatile gases produced during combustion act to reduce the possibility of combustion.

**Figure 6:**
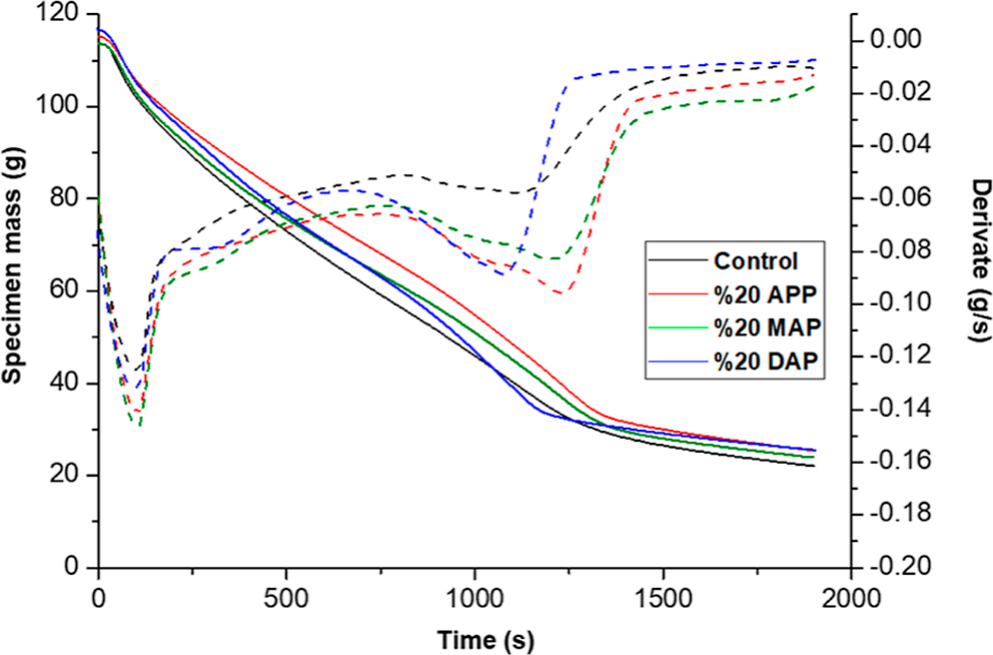
Curves of change in mass of particleboard samples over time and derivatives of these curves.

Another parameter that can be quantified by a cone calorimeter is the mass loss rate. Particleboard material has a greater FR capacity when its mass loss values are lower, and its coal residue content is higher during combustion. When the results of the mass loss rate values of the samples were examined, it was shown that the lowest value was observed in the sample containing 20% APP chemical. The results obtained from the 20% MAP and 20% DAP samples were found to be highly comparable to the results obtained from the control sample. A graphical representation, Figure 6, demonstrates the change in mass of the particleboard samples over time.

**Figure 7:**
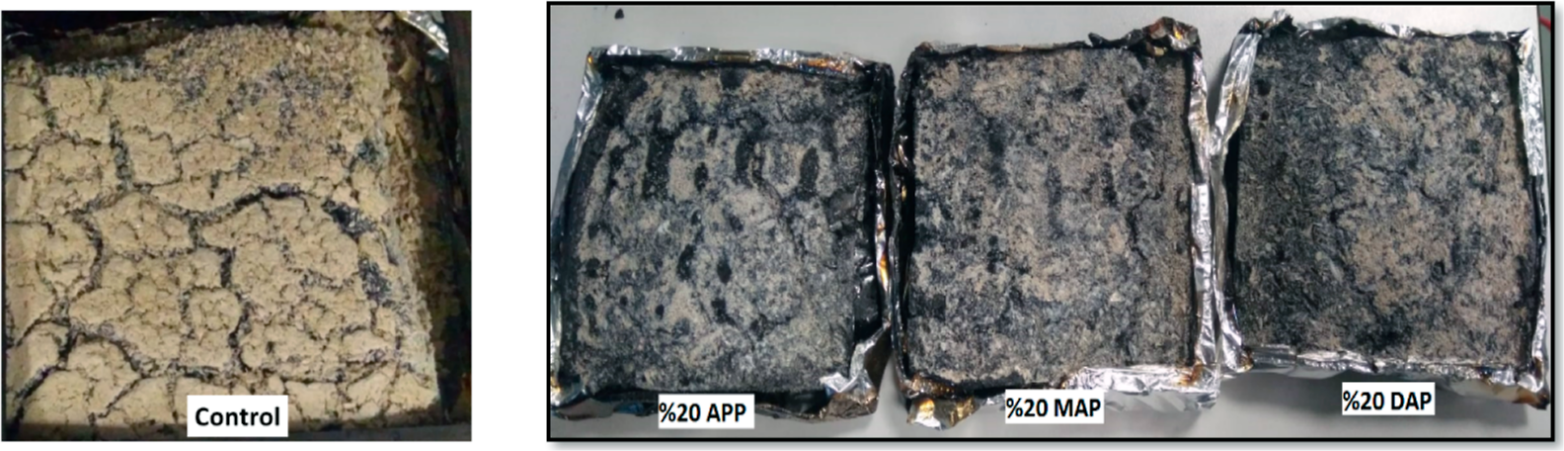
Image of the remaining samples after cone calorimetry analysis.

The results of the analysis of the mass loss rate of the particle board show that the sample with 20% APP has the lowest loss rate. As illustrated in Figure 7, the residual samples resulting from the analysis are depicted. When images of residue samples are examined, the sample containing 20% APP, which has a lower mass loss than the control sample, has a less brown structure, and the appearance of charring is more pronounced.

### 3.6 Scanning Electron Microscopy

**Figure 8:**
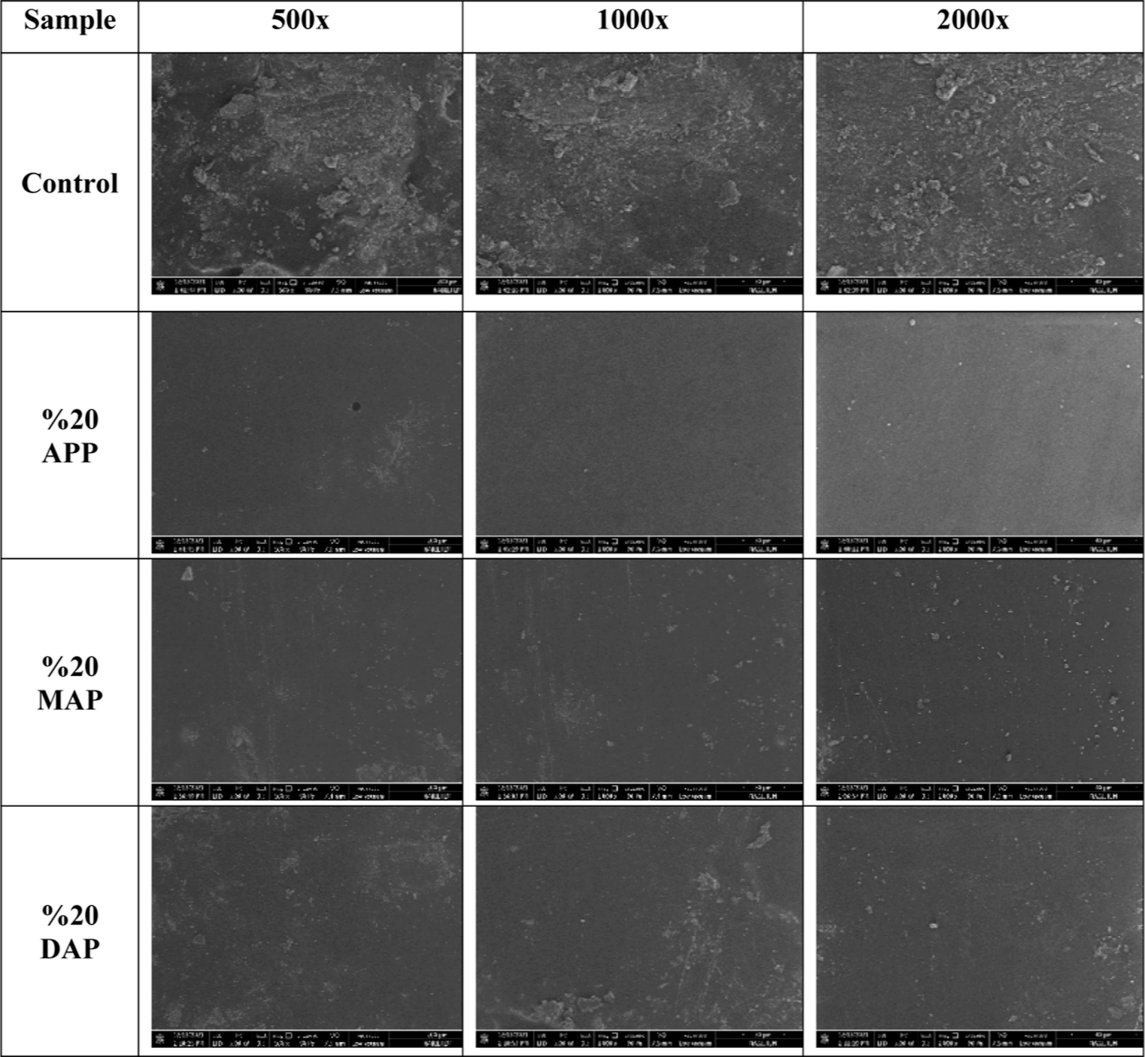
SEM images of particle boards coated with control and FR resin.

The surface morphologies of the prepared samples were examined by SEM and EDX. SEM images of all samples are given in Figure 8. With the addition of the FR, it is seen that a smoother image is formed on the surfaces, in comparison to the control sample. In particular, the sample with the smoothest and most homogeneous surface image was seen with 20% APP added.

**Figure 9:**
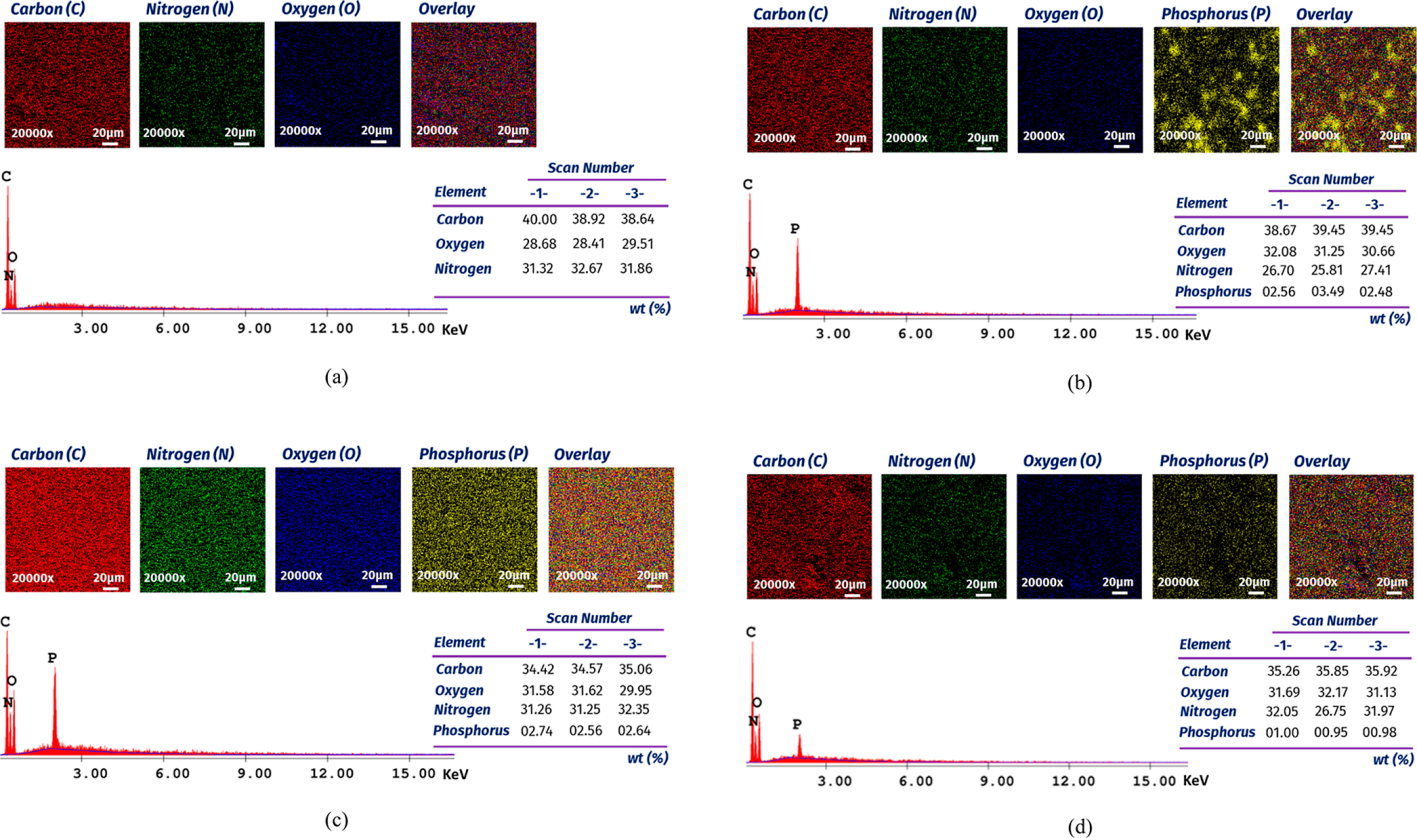
SEM–EDX elemental analysis results of (a) control, (b) 20% APP, (c) 20% MAP, and (d) 20% DAP.

Figure 9 shows the EDX spectrum and atomic composition of the selected mixtures. It is seen that there were no traces of phosphorus (P) atoms observed in the control sample. However, with 20% APP, 20% MAP, and 20% DAP, the P amount was obtained as 2.84, 2.65, and 0.98%, respectively. The EDX mapping approach was utilized to explore the dispersion of the fire retardant in resin. The method identified P-rich regions with yellow color, while the remaining matrix was black.

## 4 Conclusions

This study aimed to increase both the gelation time of the resin and the flame resistance of the particleboard by adding phosphate-containing chemicals in different proportions to the 65% UF resin used in the production of particleboard. When the FR was added, the pH of the resin decreased and gelation was achieved in the resin. No gelation was observed in resins with only 15% and 20% DAP added. Both control and resins with 10% and 20% FR added were cured by keeping them in an oven, and then, TGA was performed. According to the analysis result, the amount of substance remaining without decomposition was also in the sample containing, at most, 20% APP.

In the results of the UL-94 vertical combustion test, the control sample, that is, the particleboard sample without the FR chemicals, burned for more than 4 min. Thus, it has entered the category of flammable substances. Particleboard samples with added FR chemicals entered the V-0 grade. According to the LOI results, it was observed that particleboard samples coated with 20% FR chemically added resin had the highest flame resistance with LOI values of 29.1% and 29.2%. In comparison to the control sample, a 16% increase in the LOI values was observed. This result shows that the added FR chemicals enhance the flame resistance compared to that of the control sample, and it can be said that the study achieved the desired goal. A cone calorimetry test was performed on the samples containing 20% APP, MAP, and DAP. When the results are examined in comparison to the control sample; a 50% enhancement was found in the ignition time of the particleboard sample containing 20% APP. In other words, the sample containing 20% APP has the highest ignition time and thus a higher fire resistance.

The MARHE value decreased from 176.5 kW/m^2^ for the control sample to 120 kW/m^2^ when 20% APP was added. At the same time, the total heat dissipation value in the 0–300 s range decreased when the FR was added compared to that of the control, and 20% APP addition provided the highest reduction. When the images of the residue samples obtained after the particleboard sample combustion are examined, it was observed that the brown structure was less in the sample containing 20% APP, which has a low mass loss compared to the control sample, and the carbonization image was more pronounced: a structure with more coal layers was formed. When all of the analysis results were evaluated, the physical properties of the particle board samples gave results in the desired ranges. According to the results of thermal and morphological analyses, the sample containing 20% APP gave the best results. At the same time, it is seen that the fire-retardant effect of UF resin containing 20% APP gives results such as a high LOI value for fire performance, a V-0 class in the UL-94 vertical combustion test, and a lower MARHE value in cone calorimetry results.
